# Early Shifts of Brain Metabolism by Caloric Restriction Preserve White Matter Integrity and Long-Term Memory in Aging Mice

**DOI:** 10.3389/fnagi.2015.00213

**Published:** 2015-11-13

**Authors:** Janet Guo, Vikas Bakshi, Ai-Ling Lin

**Affiliations:** ^1^Sanders-Brown Center on Aging, University of Kentucky, Lexington, KY, USA; ^2^Department of Pharmacology and Nutritional Sciences, University of Kentucky, Lexington, KY, USA; ^3^Department of Biomedical Engineering, University of Kentucky, Lexington, KY, USA

**Keywords:** caloric restriction, neuroimaging, glucose metabolism, ketone bodies, creatine, white matter integrity, long-term memory, brain aging

## Abstract

Preservation of brain integrity with age is highly associated with lifespan determination. Caloric restriction (CR) has been shown to increase longevity and healthspan in various species; however, its effects on preserving living brain functions in aging remain largely unexplored. In the study, we used multimodal, non-invasive neuroimaging (PET/MRI/MRS) to determine *in vivo* brain glucose metabolism, energy metabolites, and white matter structural integrity in young and old mice fed with either control or 40% CR diet. In addition, we determined the animals’ memory and learning ability with behavioral assessments. Blood glucose, blood ketone bodies, and body weight were also measured. We found distinct patterns between normal aging and CR aging on brain functions – normal aging showed reductions in brain glucose metabolism, white matter integrity, and long-term memory, resembling human brain aging. CR aging, in contrast, displayed an early shift from glucose to ketone bodies metabolism, which was associated with preservations of brain energy production, white matter integrity, and long-term memory in aging mice. Among all the mice, we found a positive correlation between blood glucose level and body weight, but an inverse association between blood glucose level and lifespan. Our findings suggest that CR could slow down brain aging, in part due to the early shift of energy metabolism caused by lower caloric intake, and we were able to identify the age-dependent effects of CR non-invasively using neuroimaging. These results provide a rationale for CR-induced sustenance of brain health with extended longevity.

## Introduction

Brain integrity plays a major role in determining lifespan and cognitive status (Mattson et al., 2002). There is a strong positive correlation between brain anatomical size and maximum lifespan among mammalian species (Hofman, 1983; Bartke et al., 1998). Modulating neuroendocrine systems, such as insulin-like growth factor as well as glucose homeostasis, have shown to increase lifespan in various mouse models (Bartke et al., 1998). This is consistent with literature that brain glucose metabolism plays a significant role in sustaining neuronal viability, brain structural integrity, and consequently cognitive functionality (Bauernfeind et al., 2014). Failure to maintain cerebral metabolic rate of glucose (CMR_glc_) has shown to lead to cognitive impairment and brain volume atrophy as observed in normal aging, as well as in patients with diabetes and Alzheimer’s disease (AD; Everson-Rose and Ryan, 2015). Collectively, these suggest that preserving brain metabolism, anatomical integrity, and cognition are critical for maximizing lifespan and healthspan.

Caloric restriction (CR), without malnutrition, has been repeatedly shown to extend life expectancy and increase healthspan in various organisms (Redman et al., 2008; Colman et al., 2009; Choi et al., 2011; Rahat et al., 2011). In the peripheral system, CR dramatically improves glucose homeostasis and insulin sensitivity (Larson-Meyer et al., 2006; Baumeier et al., 2015). In the central nervous system, CR upregulates brain-derived neurotrophic factor (Lee et al., 2000, 2002; Thrasivoulou et al., 2006), reduces oxidative stress and inflammation (Gong et al., 1997; Sreekumar et al., 2002; Merry, 2004; Agarwal et al., 2005), and improves memory and learning (Pitsikas et al., 1990; Pitsikas and Algeri, 1992; Means et al., 1993). In line with this, mice treated with CR had lower incidences of age-related neurodegenerative disorders and diabetes (Park et al., 2005; Duan and Ross, 2010). However, little is known as regard to the effects of CR on *in vivo* CMR_glc_, structural integrity, and cognitive functions in the context of brain aging.

In this study, our goal was to use non-invasive neuroimaging as biomarkers to identify the impact of caloric intake on brain integrity over time. Specifically, using a long-lived mouse model, we wanted to understand whether CR-induced increased longevity could be reflected by brain functions among young and old mice. We used multi-metric imaging methods (PET/MRI/MRS) to determine CMR_glc_, brain metabolites and structural connectivity, and identified associations of their changes with cognitive functions. We hypothesized that CR would slow down brain aging by modulating brain metabolic, structural, and cognitive functions in aging mice.

## Materials and Methods

### Experimental Design

**Figure 1:**
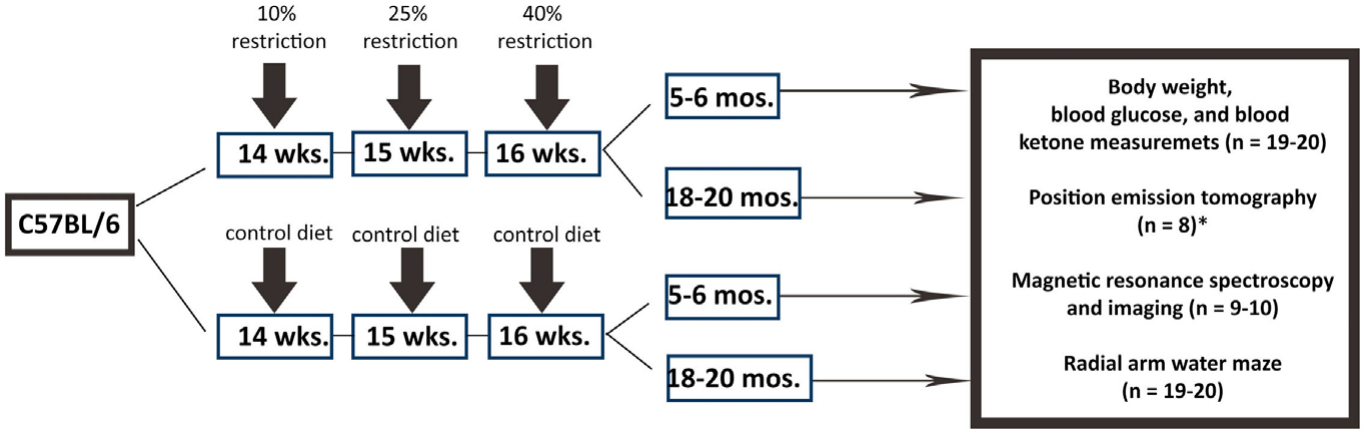
**Experimental design**. We used C57BL/6 mice in the study. Male mice were obtained from the National Institute on Aging (NIA) Caloric Restriction Colony. At NIA, all mice were fed *ad libitum* until 14 weeks of age. The CR regimen was initiated by incremental caloric reduction of 10% at week 14, 25% at week 15, and reaching full 40% CR by week 16 and continue the diet over the lifetime. Young CR (5–6 months of age), old CR (18–20 months of age), young control (*ad libitum*; 5–6 months of age), old control mice (*ad libitum*; 18–20 months of age) were then purchased from NIA for the study. The number of mice per group and the types of experimental procedure are indicated. *PET imaging was done in the University of Texas Health Science Center at San Antonio with a separate set of mice.

Figure 1 shows the overall experimental design and study timeline. We used male C57BL/6 mice in the study as they demonstrated extended longevity with CR (Forster et al., 2003; Sohal et al., 2009). Young control (5–6 months), young calorie-restricted (5–6 months), old control (18–20 months), and old calorie-restricted mice (18–20 months) were obtained from the National Institute on Aging Caloric Restriction Colony. At the National Institute on Aging, all mice were fed *ad libitum* [National Institutes of Health (NIH)-31 diet] until 14 weeks of age. The CR regimen was initiated by incremental caloric reduction of 10% at week 14, 25% at week 15, and reaching full 40% CR by week 16 and continue the diet over the lifetime. The vitamin-fortified NIH-31 (NIH-31 fortified) diet fed to CR mice provided 60% of the calories and additional vitamins supplement consumed by *ad libitum* (control) mice. After arriving at our facilities, mice were housed individually (1 mouse per cage) in a specific pathogen-free facility. The CR mice fed a pallet of CR diet between 7 a.m. and 9 a.m. everyday. Body weight was measured once a week.

We determined the sample size with power analysis in order to perform the comparison at a 0.05 level of significance, with a 90% chance of detecting a true difference of all the measurements between the four groups. Nineteen to 20 mice per group were used in the study. All the mice went through behavioral assessments. A subset of mice (*n* = 8–10 per group) was used for neuroimaging. Blood glucose and ketone bodies were measured for all the mice after sacrifice. All experimental procedures were approved by the Institutional Animal Care and Use Committee (IACUC) at the University of Kentucky (UK) according to NIH guidelines.

### Brain Structural and Metabolic Integrity Determination Using MRI

Brain structural and metabolic integrity were measured using a 7T Clinscan MR scanner (Siemens, Germany) at the Magnetic Resonance Imaging & Spectroscopy Center of UK. Mice were anesthetized with 4.0% isoflurane for induction and then maintained in a 1.2% isoflurane and air mixture using a facemask. Heart rate (90–130 bpm.), respiration rate, and rectal temperature (37 ± 0.5°C) were continuously monitored. A water bath with circulating water at 45–50°C was used to maintain the body temperature.

White matter structural connectivity in corpus callosum (CC) was used as an index for brain structural integrity. CC, connecting the right and left cerebral hemispheres of the brain, is known with a decrease in size with age (Ota et al., 2006). We used MRI-based diffusion tensor imaging (DTI) to measure fractional anisotropy (FA) in CC. The images were acquired using four-segment, spin-echo, echo-planar sequence with the following parameters: field of view = 19.2 mm × 19.2 mm, 160 × 160 matrix, slice thickness = 0.5 mm, slice numbers = 6, repetition time (TR) = 1400 ms, echo time (TE) = 42 ms, 90 degree flip angle, *b* value = 0 and 800 s/mm^2^, diffusion direction = 106, diffusion gradient amplitude (G) = 10 and 190 mT/m, gradient duration (Δ) = 18 ms, and averages = 16. The measured water diffusion was fitted to a simple tensor model with a 3 × 3 symmetrical matrix, from which three eigenvalues (λ_1_, λ_2_, λ_3_) and corresponding eigenvectors (ν_1_, ν_2_, and ν_3_) could be determined (Oishi et al., 2011). FA was then computed by the following equation (Shu et al., 2013):

(1) $$\text{FA}=\sqrt{\frac{1}{2}}\frac{\sqrt{{\left(\lambda_{1}-\lambda_{2}\right)}^{2}+{\left(\lambda_{2}-\lambda_{3}\right)}^{2}+{\left(\lambda_{3}-\lambda_{1}\right)}^{2}}}{\sqrt{\lambda_{1}^{2}+}\lambda_{2}^{2}+\lambda_{3}^{2}}.$$

Following DTI, brain metabolite levels were determined with proton (^1^H) MR spectroscopy (MRS) using a point-resolved spectroscopy sequence. Water-suppressed spectra were acquired with following parameters: TR = 1500 ms, TE = 135 ms, spectral width = 60 Hz, and average = 400. A voxel of interest of 13 mm^3^ (2.0 mm × 5.0 mm × 1.3 mm) covered bilateral hippocampus. An acquisition of non-water-suppressed spectrum with 10 averages was followed (the rest of the parameters were kept the same). Both with- and without-water suppression spectra were then processed using LCModel, which included the basis set: alanine (Ala), total choline (TCho), glutamate-glutamine complex (Glx), myo-inositol (mI), lactate (Lac), N-acetyl aspartate (NAA), phosphocreatine (PCr), total creatine (TCr), and taurine (Tau) (Provencher, 1993; Oz et al., 2010). Quantitative concentrations of the metabolites (in micromolar) were computed by Eq. 2:

(2) $$[m]=\left(\frac{S_{\text{m}}}{S_{\text{water}}}\right)[\text{water}]C_{\text{n}}C_{\text{av}}$$

where [*m*] is the concentration of the metabolite under investigation, *S*_m_ is the metabolite intensity obtained from MRS, *S*_water_ is the water intensity obtained from MRS, and [water] is the water concentration (55.14 mM at 310 K), *C*_n_ is the correction for the number of equivalent nuclei for each resonance, and *C*_av_ is the correction for the number of average (Graaf, 2007).

### Cerebral Metabolic Rate of Glucose (CMR_glc_) Measurement Using PET

We used fluorodeoxyglucose (^18^FDG) positron emission tomography (PET) to measure CMR_glc_ (Focus 220 microPET, Siemens, Nashville, TN, USA). The PET experiments were conducted at the Research Imaging Institute of the University of Texas Health Science Center at San Antonio (UTHSCSA). The experimental procedure was approved by the IACUC of UTHSCSA. A separate cohort of mice (*N* = 8 per group) was used in the PET experiments. A quantity of 0.5 mCi of ^18^FDG dissolved in 1 mL of physiologic saline solution was injected through the tail vein. Forty minutes were allowed for ^18^FDG uptake before scanning. Animals were then moved to the scanner bed and placed in the prone position. Emission data were acquired for 20 min in a 3-dimensional (3D) list mode with intrinsic resolution of 1.5 mm. For image reconstruction, 3D PET data were rebinned into multiple frames of 1-s duration using a Fourier algorithm. After rebinning the data, a 3D image was reconstructed for each frame using a 2D-filtered back projection algorithm. Decay and dead time corrections were applied to the reconstruction process. CMR_glc_ was determined using the mean standardized uptake value (SUV) equation: SUV (A × W)/Ainj, where A was the activity of the region of interest (i.e., brain region in the study), W was the body weight of the mouse, and Ainj was the injection dose of the ^18^FDG, as described in a previous study (Pulliam et al., 2014).

### Behavioral Assessments

The behavioral assessments were performed in the Rodent Behavioral Core of UK. We used the radial arm water maze (RAWM) task to measure both spatial working memory (Arendash et al., 2001; Sood et al., 2007) and spatial reference memory. Briefly, the maze consisted of six arms 160 cm in diameter with arm length 30 cm and common circular swim area of 40 cm. The pool was filled with water, dyed with non-toxic tempera paint, until the level was ~2 cm above (covering) a clear (invisible) 10-cm circular platform. The platform was placed in the back of an arm ~7 cm away from the side and back walls. The pool was located in the center of a room and enclosed by a black curtain. Geometric extra-maze visual cues were fixed throughout the study on three sides of the curtains.

The RAWM protocol consisted of a 2-day testing paradigm. A staggered training schedule was used, running the mice in cohorts of 10 mice, while alternating the different cohorts through the trials over day 1 and day 2 of the test. This alternating protocol was used to avoid the learning limitations imposed by massed subsequent trials and to avoid fatigue that may result from consecutive trials. During Block 1 (six trials) and Block 2 (six trials), mice were trained to identify the platform location by alternating between a visible and a hidden platform in the goal arm, with three hidden platform trials and three visible platforms. Block 3 consisted of three trials all with a hidden platform. These first three blocks tested spatial working memory since the mice were forced to use their short-term memory to identify the arm that contained the platform. During day 2, mice were tested in three additional blocks, all consisting of five trials using only the hidden platform (15 total trials). These second three blocks tested spatial reference memory since the mice were forced to use their spatial memory after a 24-h retention period in order to locate the platform. The mouse was released from a different start arm during each subsequent trial and allowed to identify the constant platform location. Once the platform was reached, the mouse was allowed to remain on it for 10 s before it was removed, dried, and placed in its home cage on a heating pad.

The mouse performance was recorded by EthoVision XT 8.0 video tracking software (Noldus Information Technology). Every arm entry for each mouse was recorded and reviewed to ensure that the mice did not employ non-spatial strategies, such as chaining, to solve the RAWM task. Data are presented as the average errors per block; an error was defined as when the mouse’s entire body entered an arm not containing the platform. Only errors during the hidden platform trials are included in analysis to properly represent the spatial memory of the mice.

### Blood Glucose and Ketone Bodies Measurements

When the mice were sacrificed, blood sample was collected in 500 μl lithium heparin 12.5 IU Terumo Capiject Capillary blood collection tubes (Vacutainer K2 EDTA) to avoid blood coagulation. A total of 1–2 μl of blood sample were used to measure blood glucose level using a blood glucose meter and a test strip (Clarity Plus, Boca Raton, FL, USA). Another 10 μl of blood sample was used for ketone bodies level measurement using a STAT-Site M (β-Hydroxybutyrate) meter and a test strip (Standbio Ketosite STAT-Site M-β HB, Boerne, TX, USA).

### Statistical Analysis

Statistical analyses were performed using GraphPad Prism (GraphPad, San Diego, CA, USA). We used two-way analysis of variance (ANOVA) to determine diet, age, and diet × age effects on the measured variables. Tukey’s test was further used as a *post hoc* test to detect between-group differences. Values of *p* < 0.05 were considered statistically significant.

## Results

### Caloric Restriction Induced Early Onset of Glucose Reduction and Ketone Bodies Increase

**Figure 2:**
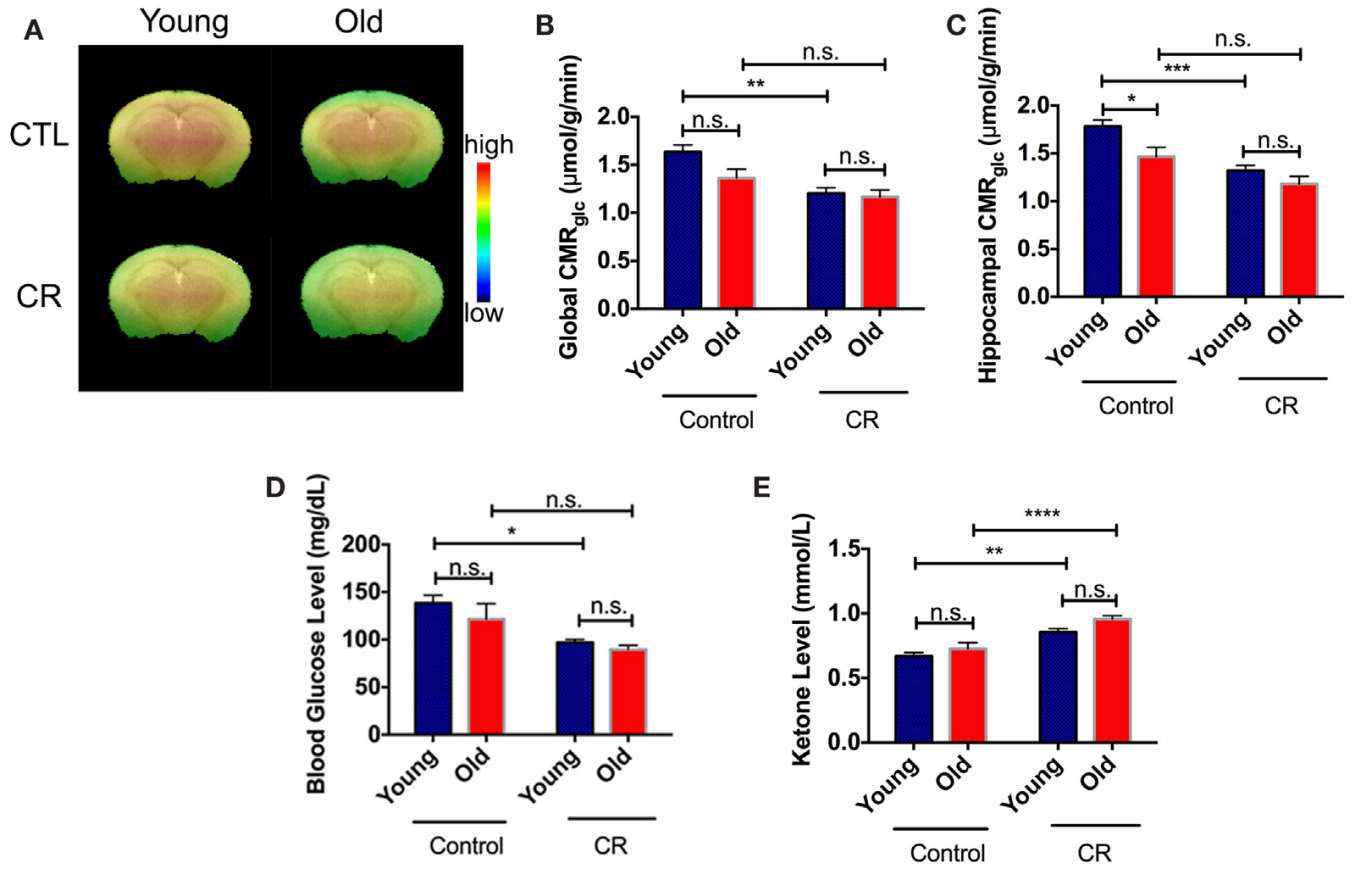
**Caloric restriction induced early onset of glucose reduction and ketone bodies increase**. **(A)** CMR_glc_ visual map of the four mice groups. The color code indicates the CMR_glc_ (in SUV) in a linear scale. **(B)** Quantitative global CMR_glc_. **(C)** Quantitative hippocampal CMR_glc_. **(D)** Blood glucose. **(E)** Blood ketone bodies. Data are presented as Mean ± SEM. *p < 0.05, **p < 0.01, ***p < 0.001, and ****p < 0.0001.

Figure 2A shows CMR_glc_ maps of the four groups of mice obtained from PET imaging. The color code indicates the glucose uptake level (in SUV) in a linear scale. Figure 2B shows the corresponding CMR_glc_ values in the whole brain. Old control exhibits an apparent decline in global CMR_glc_ when compared to young control, as is common of the aging process (Petit-Taboue et al., 1998; Lin and Rothman, 2014). By contrast, young CR showed an initial decrease in global CMR_glc_ that is sustained in the old CR global CMR_glc_. Both diet and age had significant effects on global CMR_glc_ [diet: *F* (1,36) = 17.62, *p* = 0.002; age: *F* (1,36) = 4.272, *p* = 0.046]. Similar CMR_glc_ pattern was observed in the hippocampus, a region associated with cognitive functions (e.g., memory and learning) (Figure 2C). Diet and age also had significant effects on hippocampal CMR_glc_ [diet: *F* (1,36) = 23.81, *p* < 0.0001; age: *F* (1,36) = 8.94, *p* = 0.005]. Collectively, these results suggest that there was an early onset of CMR_glc_ reduction in the CR mice, and CR eliminated the age-dependent decline in global and regional CMR_glc_.

We had similar findings in the peripheral system. Blood glucose was significantly lower in the young CR mice levels, compared to the control groups, and remained constant with age (Figure 2D). We found significant effects of diet [*F* (1,34) = 16.4, *p* = 0.0003], but not age [*F* (1,34) = 1.776, *p* = 0.1915], on the blood glucose levels. Alternatively, CR was shown to increase in ketone bodies of CR mice compared to control mice (Figure 2E). Both diet and age had significant effects on blood ketone bodies [diet: *F* (1,34) = 40.93, *p* < 0.0001; age: *F* (1,34) = 5.962, *p* = 0.02].

### Caloric Restriction Increased Production of Brain Energy Metabolite and Preserved it with Age

**Figure 3:**
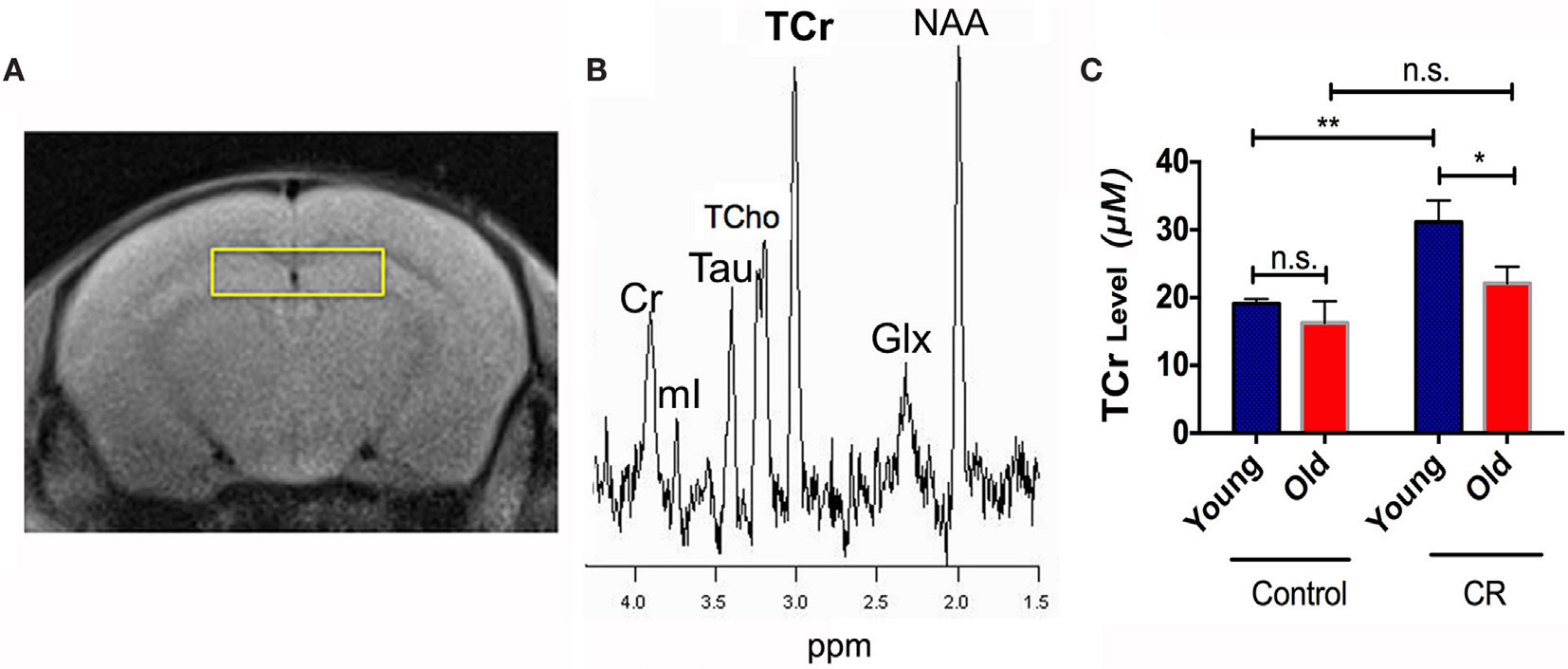
**Caloric restriction increased production of brain energy metabolite and preserved it with age**. **(A)** The voxel replacement on the hippocampus and **(B)** the representative ^1^H-MRS spectrum, showing total choline (TCho), total creatine (TCr), taurine (Tau), glutamate-glutamine complex (Glx), myo-inositol (mI), *N*-acetylaspartate (NAA), in parts per million (ppm). **(C)** TCr levels of the four groups. Data are presented as Mean ± SEM. *p < 0.05; **p < 0.01; n.s., non-significant.

Figure 3A shows the voxel replacement on bilateral hippocampus for MRS experiments. Figure 3B demonstrates a representative spectrum of ^1^H-MRS, showing peaks of NAA, Glx, TCr, Cho, and mI. Table 1 shows the quantitative results of the major metabolites. We found significant diet and age effects on TCr [diet: *F* (1,30) = 11.60, *p* = 0.0019; age: *F* (1,30) = 5.121, *p* = 0.031]. Figure 3C shows that young CR mice had significant increases of TCr compared to young control mice. Although TCr dropped dramatically when the CR mice were getting old, the level was still comparable to the young controls (*p* = 0.1596), and higher than that of the old controls (*p* < 0.05). TCr is the sum of creatine and PCr (crucial role as an intracellular buffer during the production of ATP) (Wallimann et al., 2011), suggesting that CR increases ATP production in young CR mice, and preserves ATP production in old CR mice, relative to controls. We also found significantly elevated Tau in young CR compared to the young control mice (*p* < 0.05; Table 1). As Tau is associated with neurotransmitter modulation, this may indicate that young CR mice have an early enhancement of neuronal activity compared to the age-matched controls (Makarova et al., 2014; Xu et al., 2015).

**Table 1:** **Major MRS metabolites quantitation**.

Metabolites	Control		CR	
	Young	Old	Young	Old
Ala	3.0 ± 0.4	1.3 ± 0.5	3.3 ± 1.0	2.3 ± 0.5
Glx	26.1 ± 2.9	30.1 ± 6.1	36.9 ± 2.1	27.5 ± 3.4
Lac	7.2 ± 0.9	6.7 ± 1.3	5.7 ± 2.0	4.5 ± 0.3
mI	15.4 ± 3.9	18.9 ± 3.7	18.9 ± 4.0	16.0 ± 2.4
NAA	45.6 ± 2.7	53.1 ± 5.2	56.7 ± 2.0	55.8 ± 5.8
PCr	29.1 ± 2.1	36.9 ± 4.3	29.1 ± 3.6	33.2 ± 4.6
Tau	39.7 ± 4.2^a^	45.8 ± 5.1	55.5 ± 2.1	42.9 ± 3.3
TCho	11.5 ± 1.1	13.1 ± 0.7	12.3 ± 0.9	11.6 ± 0.6
TCr	19.1 ± 0.7^a^	16.3 ± 3.2^b^	31.2 ± 3.2^c^	22.1 ± 2.4

*Data are presented as mean ± SEM in micromolar*.

*^a^Indicates a statistical difference between young control and young CR*.

*^b^Indicates a statistical difference between old control and old CR*.

*^c^Indicates a statistical difference between young CR and old CR*.

*Ala, alanine; Glx, glutamate-glutamine complex; Lac, lactate; mI, myo-inositol; NAA, N-acetyl aspartate; PCr, phosphocreatine; Tau, taurine; TCho, total choline; TCr, total creatine*.

### Caloric Restriction Preserved White Matter Structural Integrity

**Figure 4:**
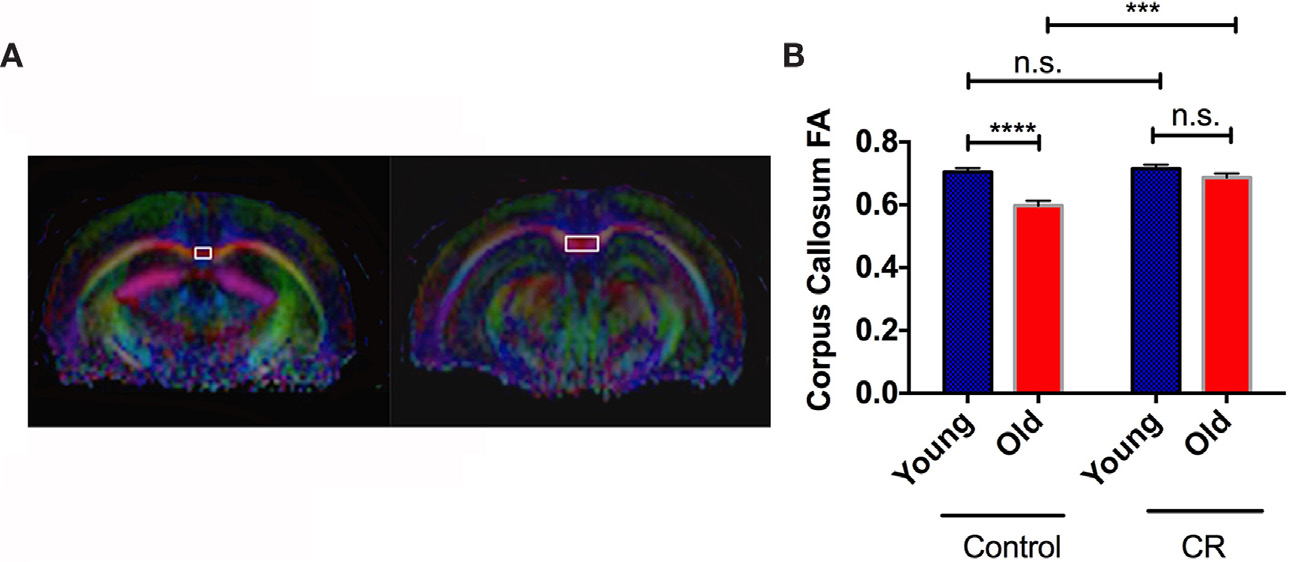
**Caloric restriction preserved white matter structural integrity**. **(A)** The region showing corpus callosum (CC) on MRI diffusion-weighted images. **(B)** The quantitative measurements of fractional anisotropy (FA) in CC. Data are presented as Mean ± SEM. ***p < 0.001 and ****p < 0.0001.

Figure 4A shows the representative color-coded diffusion-weighted images. CC was indicated by the white boxes. We found significant diet, age, and diet × age effects on FA values of CC [diet: *F* (1,36) = 13.64, *p* = 0.0007; age: *F* (1,36) = 24.37, *p* < 0.0001; diet × age: *F* (1,36) = 8.345, *p* = 0.0065]. Figure 4B shows that old control had significantly less CC FA than young control, similar to the findings in human aging (Kochunov et al., 2011). However, we did not see the age-dependent decline of FA in the CR groups, indicating preserved CC integrity with age by CR.

### Caloric Restriction Prevented Age-Dependent Long-Term Memory Deterioration

**Figure 5:**
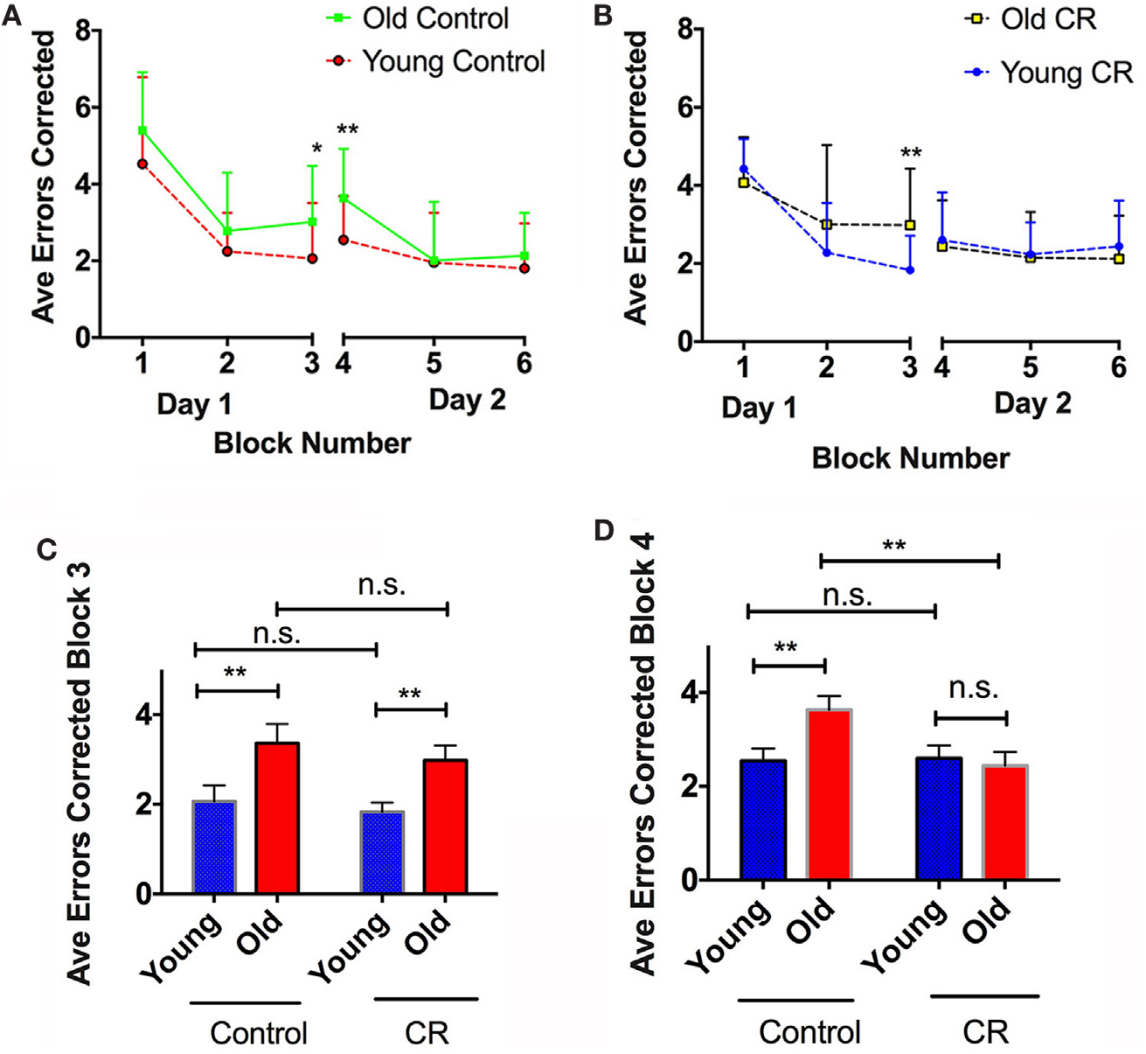
**Caloric restriction prevented age-dependent long-term memory deterioration**. **(A)** Average errors corrected over six blocks of the control mice, with significant cognitive difference observed on Blocks 3 and 4. **(B)** Average errors corrected over six blocks of the CR mice, with significant cognitive difference observed on Block 3. **(C)** Comparison of the errors made by the four groups on Block 3, an index of learning ability and short-term memory. **(D)** Comparison of the errors made by the four groups on Block 4, and index of long-term memory. Data are presented as Mean ± SEM. *p < 0.05; **p < 0.01.

Figures 5A shows the average errors made by the control mice during the two-day RAWM testing. The old control mice overall made more errors than the young controls in Day 1, reaching a significant difference in Block 3 when the visible platform was removed (Figure 5C). This indicates that old control mice had less ability to learn new task and retain short-term memory. The old control mice also committed significantly more errors than the young controls in Block 4 (Day 2) (Figure 5D), indicating that old mice had difficulty to retain long-term memory compared to the young mice. By contrast, old CR mice did not show significant differences in RAWM performance compared to the young CR, except in Block 3 (Figures 5B,C). This indicates that old CR mice might have more difficulty to learn a new task; however, once they learned, they remembered it well, as evident by the similar performance in Block 4 (Figure 5D). Taken together, our results suggest that CR may be able to impede long-term memory deterioration often found in the aging process.

### Association of Caloric Restriction with Blood Glucose, Body Weight, and Lifespan

**Figure 6:**
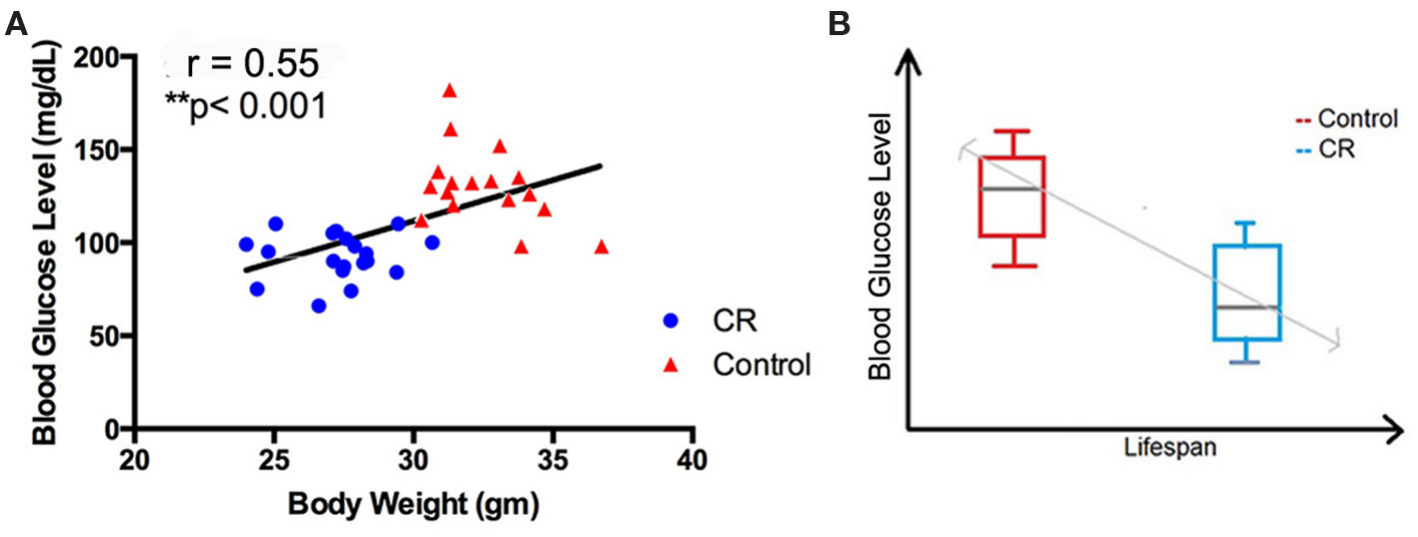
**Association of caloric restriction with blood glucose, body weight, and lifespan**. **(A)** A positive correlation between blood glucose and body weight among the CR and control mice (*r* = 0.55, *p* < 0.001). **(B)** An inverse association between blood glucose level (measured in the current study) and the lifespan [reported by Forster et al. (2003)] of the CR and control mice.

Using linear regression, we observed a significant correlation between blood glucose level and body weight among the four groups of the mice (*r* = 0.55; *p* < 0.001). Control mice, regardless of age, had higher blood glucose and body weight, compared to the CR mice (Figure 6A; Table 2). In addition, we observed an inverse association between blood glucose and reported longevity among the control and CR mice (Figure 6B; Table 2).

**Table 2:** **Age, body weight, median lifespan, and maximum lifespan of the mice**.

	Age (months)	Body weight (grams)	Median lifespan (months)^a^	Maximum lifespan (months)^a^
Young control	5–6	31.93 ± 0.37	26.3	34.3
Old control	18–20	33.96 ± 1.01		
Young CR	5–6	26.67 ± 0.66*	32.6	42.7
Old CR	18–20	27.94 ± 0.24**		

*Young CR < Young control (*p* < 0.0001); **Old CR < Old control (*p* < 0.0001). Data are presented as Mean ± SEM.

*^a^Data reported from Forster et al. (2003)*.

## Discussion

In the study, we used neuroimaging and behavioral tests to demonstrate significant dietary effects on brain functions with age. There are five key findings from the studies reported here. First, there was an early onset of glucose reduction induced by CR. By contrast, increased ketone bodies were found in the young CR mice. These changes were age-independent – old CR mice had similar levels on those indices when compared to their young littermates. The findings are consistent with the literature, indicating CR induced a metabolic shift from utilizing glucose to ketone bodies (Shimazu et al., 2013; Lin et al., 2015). Second, we found that CR increases ATP production in young CR mice and preserves ATP production in old CR mice, relative to controls, based on the TCr data; young CR mice had significantly higher Tau relative to young control, indicating CR-induced higher neuronal activity in the young mice. Third, we observed a preservation of white matter structural integrity with age in the CR mice. Fourth, the mice fed with chronic CR diet preserved long-term memory. Finally, we found associations between glucose level, body weight, and lifespan. Taken together, we observed distinct patterns between normal aging and CR aging on brain functions. Normal aging shows reductions in brain glucose metabolism, white matter integrity, and long-term memory, resembling human brain aging. CR aging, in contrast, displays early onset changes in brain metabolism and neuronal activity, and preservations of energy production, white matter integrity, and long-term memory in aging mice.

Our findings suggest that the benefits found in the CR mice might be in part due to the early shift of energy metabolism caused by lower caloric intake. Because of reduced glucose availability, CR mice adapted to use ketone bodies metabolism at a very early age. These metabolic alterations remained stable with age. Moderate ketosis has been shown to have many beneficial properties for brain functions, including sustaining neuronal activities (Masino et al., 2009), preserving energy substrates (Sullivan et al., 2004), enhancing memory (Nordli et al., 2001; Pulsifer et al., 2001), reducing insulin resistance, and alleviating damage caused by oxidative stress and hypoxia (Cahill and Veech, 2003; Sullivan et al., 2004; Maalouf et al., 2007). However, whether feeding ketogenic diet will mimic CR’s beneficial effects on brain functions remains an object of study (Brownlow et al., 2013).

We observed enhanced TCr (suggesting increased ATP production) in the young CR mice and preserved TCr as well as long-term memory in the old CR mice. These observations are consistent with our previous findings that show CR preserves mitochondrial functions and ATP production in rats (Lin et al., 2014), and maintaining mitochondrial functions is critical for sustaining cognition (Fontan-Lozano et al., 2008; Mattson, 2010; Lin et al., 2013a).

Our neuroimaging and behavioral results are consistent with pervious molecular-based findings. These CR-induced changes have shown to be mediated by mammalian target of rapamycin (mTOR) and sirtuines 1 (SIRT1) signaling. Both mTOR and SIRT1 are involved in nutrient-sensing pathways. Reduced food intake, such as with the condition of CR, inhibits mTOR and activates SIRT1 (Al-Wahab et al., 2015). mTOR inhibition reduces growth signaling and enhances catabolic activity. Thus, mTOR inhibition plays an important role in mediating the switch of cellular recourses from growth and reproduction toward somatic maintenance as revealed by the increase in resistance to various stresses (Kapahi, 2010). In addition, mTOR inhibition upregulates autophagy and cerebrovascular responses, which enhance the clearance of mis-folded proteins, including beta amyloid (Aβ), a hallmark of AD (Lin et al., 2013b; Dong et al., 2015). Increased SIRT1 activity, on the other hand, reduces cellular oxidative stress and DNA damage, enhances neuroplasticity, facilitates fat loss, and shifts metabolisms away from using glucose as an energy source (Ng et al., 2015). These are consistent with our current and previous studies, showing that CR shifts from glucose to ketone bodies utilization (Lin et al., 2015), preserves mitochondrial functions (Lin et al., 2014), preserves brain structural connectivity, enhances cognitive function, and consequently increases the ability of the brain to resist aging and age-related neurodegenerative disorders (Fontan-Lozano et al., 2008).

A decrease in relying on glucose utilization might be neuroprotective in the aging brain. Recent studies have shown that brain areas that are highly dependent on glucose utilization at an early age have strong levels of Aβ deposition later in life, suggesting a correlation between high glucose utilization and Aβ deposition (Vlassenko et al., 2010). Other studies have shown that increased glucose levels in the brain are associated with neurotoxicity, brain size reduction, white matter connectivity deterioration, and cognitive impairment (Tomlinson and Gardiner, 2008; Colman et al., 2009; Mortby et al., 2013). This is consistent with the findings that animal models under CR had reduced Aβ accumulation and lower incidence to develop AD (Qin et al., 2006), and in a good agreement with our findings that CR mice had preserved white matter integrity and cognitive functions. This could have been because CR mice did not rely on glucose as the main energy source, beginning at an early age, and thus have been protected from the age-related glucose metabolism impairment, which includes reduced glucose uptake, increased glucose intolerance, increased insulin resistance, and inflammation (Fink et al., 1983; Rowe et al., 1983; Stout, 1994).

Elevated glucose levels with age, in contrast, could be detrimental and have been shown to shorten lifespan (Lee et al., 2009). Consistent with this finding, we observed an inverse association between blood glucose level and the reported lifespan of the mice (Figure 6B; Table 1). By contrast, blood glucose levels had a strong positive correlation with body weight (Figure 6A). This is in good agreement with the literature, suggesting that body weight [or body-mass index (BMI)] could be an indicator of blood glucose and that by preserving the BMI in a normal range, blood glucose might be maintained at a healthy level (Sepp et al., 2014). Collectively, we demonstrated that mice with lower caloric intake were overall healthier, with increased CNS healthspan and lifespan.

It has to be pointed out that we used a long-lived rodent model in the present study to investigate CR effects. Recent studies have shown that the lifespan response to a single level of CR (e.g., 40% CR) varies widely in mice from different genetic backgrounds (Liao et al., 2010). In some cases, CR shortened the lifespan in inbred mice. The main findings in the studies were that CR life extension correlated inversely with fat reduction – strains with the least reduction in fat were more likely to show life extension, and those with the greatest reduction were more likely to have shortened lifespan (Liao et al., 2011). As fatty acids in astrocytes are needed for ketone body metabolism, those with shorter lifespans may not be able to upregulate ketone body utilization under CR. Reduction in CMR_Glc_ without elevated ketone bodies may lead to shorter lifespan. Similarly, cognitive response to CR might also vary significantly with genetics in mice modeling human AD. Although some of the strains responded favorably to CR, others did not (Brownlow et al., 2014). These suggest that genetic factor may play a significant role in mediating the responses to dietary interventions. It will be important in the future to use neuroimaging to determine if CR also has adverse effects on brain metabolic functions in rodent strains where deleterious effects on lifespan or cognitive functions are observed.

Investigations on the effects of CR on brain functions are translatable. Previous studies show that CR can improve working memory, verbal memory, executive function memory/learning, and cognitive performance, and reduce depressive symptoms in elderly humans (Pitsikas and Algeri, 1992; Adams et al., 2008; Witte et al., 2009; Kuhla et al., 2013; Murphy et al., 2014; Rizza et al., 2014). However, how CR affects *in vivo* brain physiology remains unknown. Since the PET, MRI, and MRS used in the study are readily used in humans (Uh et al., 2011; Lin et al., 2012; Lin and Rothman, 2014), it would be important in future studies to identify CR-induced changes in metabolic and structural integrity in human brain aging using these multi-metric imaging methods.

In addition to CR, other micronutritients (e.g., vitamins B, C, D, and E, and omega-3 fatty acid) and macronutrients (e.g., fish) have also been suggested to play important roles in the prevention of cognitive decline and dementia/AD (Smith and Blumenthal, 2010; Keeney and Butterfield, 2015). In future studies, it is important to identify other nutritional effects on brain metabolic and structural integrity, as well as the involved mechanistic pathways thereof (Smith and Blumenthal, 2010; Gillette-Guyonnet et al., 2013).

One limitation of the study is the sensitivity for detecting brain metabolites using MRS. With the small brain size of the mice and the VOI on hippocampus (~13 mm^3^), we might not be able to detect the subtle changes of the several metabolites with age, including NAA, mI, and Lac (Table 1). However, we were able to measure the significant increases of TCr and Tau, which indicate robust and reliable changes of energy production and neuronal activity induced by CR.

In conclusion, we successfully used non-invasive neuroimaging to identify CR effects on brain physiology in aging mice. Specifically, we found an early shift in brain metabolism in mice with low caloric intake, which was associated with preserved energy production, brain structural integrity, and long-term memory. These findings provide a rationale for CR-induced sustenance of brain health with extended lifespan. Understanding nutritional effects on brain function may have profound implications in human aging and other age-related neurodegenerative disorders. Using multimodal neuroimaging methods, we will be in a position to identify effective nutritional interventions, and the treatment efficacy thereof to slow down brain aging and/or prevent dementia for humans.

## Author Contributions

JG, VB, and A-LL contributed to the design, acquisition, analysis, and interpretation of data for the work. JG and A-LL drafted and revised the work for important intellectual content. JG, VB, and A-LL approved of the final version and agreed to be accountable for all aspects of the work in ensuring that questions related to the accuracy or integrity of any part of the work are appropriately investigated and resolved.

## Conflict of Interest Statement

The authors declare that the research was conducted in the absence of any commercial or financial relationships that could be construed as a potential conflict of interest.
